# The Year in Cardiology 2018: ABC Cardiol and RPC at a glance

**DOI:** 10.5935/abc.20190015

**Published:** 2019-02

**Authors:** Ricardo Fontes-Carvalho, Glaucia Maria Moraes de Oliveira, Lino Gonçalves, Carlos Eduardo Rochitte

**Affiliations:** 1 Departamento de Cardiologia - Centro Hospitalar de Vila Nova de Gaia, Vila Nova de Gaia - Portugal; 2 Departamento de Cirurgia e Fisiologia - Faculdade de Medicina - Universidade do Porto, Porto - Portugal; 3 Faculdade de Medicina - Universidade Federal do Rio de Janeiro, Rio de Janeiro, RJ - Brazil; 4 Instituto do Coração Edson Saad - Universidade Federal do Rio de Janeiro, Rio de Janeiro, RJ - Brazil; 5 Departamento de Cardiologia - Centro Hospitalar e Universitário de Coimbra, Coimbra - Portugal; 6 Faculdade de Medicina - Universidade de Coimbra, Coimbra - Portugal; 7 Instituto do Coração (InCor) - Faculdade de Medicina da Universidade de São Paulo, São Paulo, SP - Brazil; 8 Hospital do Coração (HCOR), São Paulo, SP - Brazil

**Keywords:** Periodicals/trends, Scientific and Technical Activities/trends, Cardiovascular Diseases/prevention and control, Cardiovascular Diseases/epidemiology, Cardiomyopathies, Heart Valve Diseases

Portuguese is the sixth most spoken language worldwide, by 244 million speakers, the fifth most commonly used language over the Internet, by almost 83 million cybernauts, and the third most commonly used language in the social media *Facebook* and *Twitter*. Portuguese is the official language of eight countries (Portugal, Brazil, Angola, Mozambique, Guinea-Bissau, Cape Verde, Sao Tome and Principe, and Timor-Leste). Despite the incorporation of native words, and the grammatical and pronunciation changes characteristic of each country, their languages remain united and they share many important health problems such as cardiovascular diseases (CVD).^1^

Currently, two journals are published in the Portuguese language all over the world, Revista Portuguesa de Cardiologia (Rev Port Cardiol) and Arquivos Brasileiros de Cardiologia (currently nicknamed ABC Cardiol), and both published the best papers in the Portuguese language.

Rev Port Cardiol, also known as Portuguese Journal of Cardiology, is the official scientific journal of the Portuguese Society of Cardiology. With more than 35 years of uninterrupted scientific activity, it is now a prestigious international journal with global visibility.^2^

The histories of the Brazilian Society of Cardiology and ABC Cardiol have been completely interlaced since the beginning, and in 2018 ABC Cardiol completed 70 years of existence. ABC Cardiol is an open access publication, scientific home, reading for all the 14,000 cardiologists and members of the Brazilian Society of Cardiology, with almost one third of its articles coming from international authors. ABC Cardiol is indexed in the main databases and has the best Impact Factor for journals in the area of Cardiology and Cardiovascular Sciences in Latin America.^3^

Every year both journals publish dozens of high-quality scientific articles. In the year 2018, Revista Portuguesa de Cardiologia has published a total of 194 papers, with 62 original articles, and ABC Cardiol published a total of 240 papers, with 96 being original articles. The selection of the 10 best research papers (Tables 1 and 2) from both journals is always a difficult endeavor, given their overall high scientific quality. Moreover, in the absence of specific metrics, this selection is always imperfect and influenced by some degree of subjectivity. Nonetheless, a judging committee composed by highly selected scientists in the field brings us probably the fairest results for the top ten articles in these Journals. Both journals have also published several important review papers, which were out of the scope of this selection.

**Table 1:** List of the 10 best original articles published in 2018 in Revista Portuguesa de Cardiologia

Author	Title _link
Timóteo A et al.	Portuguese Registry of Acute Coronary Syndromes (ProACS): 15 years of a continuous and prospective registry https://www.sciencedirect.com/science/article/pii/S2174204918301983
Monteiro P et al.	The SAFIRA study: A reflection on the prevalence and treatment patterns of atrial fibrillation and cardiovascular risk factors in 7500 elderly subjects https://www.sciencedirect.com/science/article/pii/S2174204918300849
Pereira H et al.	Factors influencing the patient delay to primary angioplasty in myocardial infarction with ST-segment elevation (STEMI): the Stent for life initiative in Portugal https://www.sciencedirect.com/science/article/pii/S0870255117300811
Menezes MN et al.	Comparative analysis of fractional flow reserve and instantaneous wave-free ratio: Results of a five-year registry https://www.sciencedirect.com/science/article/pii/S217420491830134X
Cardim N et al.	The Portuguese Registry of Hypertrophic Cardiomyopathy: Overall results https://www.sciencedirect.com/science/article/pii/S0870255117305425
Andrade N et al.	Knowledge about cardiovascular disease in Portugal https://www.sciencedirect.com/science/article/pii/S0870255117306832
Timóteo A et al.	What is the role of beta-blockers in a contemporary treatment cohort of patients with acute coronary syndrome? A propensity-score matching analysis https://www.sciencedirect.com/science/article/pii/S217420491830388X
Fontes-Carvalho R et al.	Left atrial deformation analysis by speckle tracking echocardiography to predict exercise capacity after myocardial infarction https://www.sciencedirect.com/science/article/pii/S2174204918303520
Rodrigues PM et al.	Body adiposity is associated with risk of high blood pressure in Portuguese schoolchildren https://www.sciencedirect.com/science/article/pii/S2174204918301259
Pereira-da-Silva T et al.	Optimizing risk stratification in heart failure and the selection of candidates for heart transplantation https://www.sciencedirect.com/science/article/pii/S0870255117300641

**Table 2:** List of the 10 best original articles published in 2018 in ABC Cardiol

Author	Title - link
Nascimento BR et al.	Cardiovascular Disease Epidemiology in Portuguese-Speaking Countries: data from the Global Burden of Disease, 1990 to 2016 http://www.scielo.br/scielo.php?script=sci_arttext&pid=S0066-782X2018000600500&lng=en&nrm=iso&tlng=en&ORIGINALLANG=en
Farsky PS et al.	Persistent Inflammatory Activity in Blood Cells and Artery Tissue from Patients with Previous Bare Metal Stent http://www.scielo.br/scielo.php?script=sci_arttext&pid=S0066-782X2018001400134&lng=pt&nrm=iso&tlng=en&ORIGINALLANG=en
Borges JMDM et al.	Factors Associated with Inadequate Management of Antiplatelet Agents in Perioperative Period of Non-Cardiac Surgeries http://www.scielo.br/scielo.php?script=sci_arttext&pid=S0066-782X2018001600596&lng=es&nrm=i&tlng=en&ORIGINALLANG=en
de Souza e Silva CG et al.	Up to 15-Year Survival of Men and Women after Percutaneous Coronary Intervention Paid by the Brazilian Public Healthcare System in the State of Rio de Janeiro, 1999-2010 http://www.scielo.br/scielo.php?script=sci_arttext&pid=S0066-782X2018001600553&lng=pt&nrm=iso&tlng=en&ORIGINALLANG=en
Stephan LS et al.	Oral Anticoagulation in Atrial Fibrillation: Development and Evaluation of a Mobile Health Application to Support Shared Decision-Making http://www.scielo.br/scielo.php?script=sci_arttext&pid=S0066-782X2018000100007&lng=en&nrm=iso&tlng=en&ORIGINALLANG=en
Gripp EA et al.	Global Longitudinal Strain Accuracy for Cardiotoxicity Prediction in a Cohort of Breast Cancer Patients During Anthracycline and/or Trastuzumab Treatment http://www.scielo.br/scielo.php?script=sci_arttext&pid=S0066-782X2018000200140&lng=en&nrm=iso&tlng=en&ORIGINALLANG=en
Miyazaki Y et al.	The Role of Quantitative Aortographic Assessment of Aortic Regurgitation by Videodensitometry in the Guidance of Transcatheter Aortic Valve Implantation http://www.scielo.br/scielo.php?script=sci_arttext&pid=S0066-782X2018001400193&lng=pt&nrm=iso&tlng=en&ORIGINALLANG=en
Martins CN et al.	Mid- and Longterm Neo-Aortic Valve Regurgitation after Jatene Surgery: Prevalence and Risk Factors http://www.scielo.br/scielo.php?pid=S0066-782X2018005008104&script=sci_arttext
Silva DV et al.	Comparison of Cardiac and Vascular Parameters in Powerlifters and Long-Distance Runners: Comparative Cross-Sectional Study http://www.scielo.br/scielo.php?script=sci_arttext&pid=S0066-782X2018001800772
Rodrigues JA et al.	Physical Exercise and Regulation of Intracellular Calcium in Cardiomyocytes of Hypertensive Rats http://www.scielo.br/scielo.php?script=sci_arttext&pid=S0066-782X2018001400172

## Coronary artery disease

In 2018, Rev Port Cardiol reported the 15-year results of the Portuguese Registry of Acute Coronary Syndromes (PorACS).^4^ This is a multi-center, continuous, and ongoing registry which has already involved more than 45,000 events, from 45 centers. It is one of the largest national registries in this field, which is only exceeded by the SWEDEHEART^5^ and the MINAP^6^ registries. This article provides very important information about the epidemiology and evolution of ACS treatment. First, it demonstrates that the clinical profile of ACS patients changed little over the years, and the proportion of ST-elevation myocardial infarction (STEMI) remained stable (45%). More importantly, over the years, there has been a major improvement in the overall quality of ACS care. For example, more than 85% of STEMI patients in Portugal now receive reperfusion therapy, which is mainly performed by primary PCI (only 5.2% underwent thrombolysis). This improvement in ACS care led to a remarkable reduction of in-hospital mortality, which decreased from 6.7% in 2002 to 2.5% in 2016. Therefore, the cardiovascular community must be acknowledged for this striking achievement. Nevertheless, the work is not finished, and this study also shows important gaps in ACS care that should be addressed. Unfortunately, time-to-reperfusion has not improved sufficiently, and there is an urgent need to improve both the “patient-delay” and “system-delay” times.

In another interesting article published in 2018, Pereira H. et al.^7^ evaluated the determinants of this “patient-delay” time in the Portuguese health system in 994 patients with suspected STEMI. Although most healthcare systems focus their performance measures in the evaluation of “door-to-balloon times”,^8^ it is important to understand and address the reasons for this “patient-delay time”, which means the time from symptom onset to the first medical contact.^9^ The investigators observed that patient-delay time was too long (about 120 minutes) and identified five predictors of increased patient-delay: 1) age > 75 years; 2) symptom onset between 0:00 and 8:00 a.m.; 3) attending a primary care unit before first medical contact; 4) not calling the national medical emergency number; and 5) self-transport to the emergency department. Therefore, this article provides important information to plan more effective patient-directed campaigns that can decrease patient-delay time and improve STEMI management and prognosis.^10^

The latest 2018 myocardial revascularization guidelines have focused on the importance of hemodynamic assessment of intermediate-grade coronary artery lesions, which can be done either by FFR or iFR.^11^ iFR is a new technique to access the severity of coronary stenosis, which has the advantage of not requiring the administration of a vasodilator, such as adenosine. Two recently published randomized trials have shown comparable clinical results between these two techniques in patients with intermediate-grade stenosis.^12^^,^^13^ However, some studies have shown that there can be some inconsistencies between the two measurements.^14^ In a provocative article published in Rev Port Cardiol, Menezes et al.^15^ report their experience directly comparing FFR and iFR information in 150 patients. They have demonstrated that, in general, FFR and iFR are concordant, but in a significant proportion of cases (13%) the results differed between the two techniques. Therefore, this article is another important contributor for the ongoing discussion about the underlying mechanisms responsible for this discordance and their clinical implications.^16-18^

An issue that remains open in coronary artery disease care is higher mortality after coronary artery bypass surgery (CABG) in patients with stent. Farsky et al.^17^ evaluated inflammatory markers (LIGHT, IL-6, ICAM, VCAM, CD40, NFKB, TNFα, IFN γ) in peripheral blood cells and in coronary artery tissue obtained during CABG in patients with stent (n = 41) compared to controls (n = 26). They observed that patients with stent showed higher TNFα (p = 0.03) and lower CD40 gene expression (p = 0.01) in peripheral blood cells than controls without stent. In coronary artery samples, the TNFα protein staining was higher in patients with stent, not only in the intima-media layer (5.16 ± 5.05 vs 1.90 ± 2.27; p = 0.02), but also in the adipose tissue (6.69 ± 3.87 vs 2.27 ± 4.00; p < 0.001), which had a higher interleukin-6 protein (p = 0.04). They concluded that higher systemic levels of inflammatory markers in patients with stents may contribute to a worse clinical outcome, contributing to our better understanding of pathophysiological changes that occur in patients with coronary stents who underwent coronary artery bypass grafting.

Another challenge in coronary artery management is cardiac complications and deaths in the post-operation period of non-cardiac surgeries, mainly due to acute myocardial infarction (AMI). Antiplatelet agents are the cornerstone for primary and secondary prevention of cardiovascular events. Borges et al.^18^ conducted a cross-sectional study to assess factors associated with inadequate management of antiplatelet agents in the perioperative period of non-cardiac surgeries. The sample consisted of adult patients undergoing non-cardiac surgeries and who would use acetylsalicylic acid (aspirin) or clopidogrel (n = 161). The management failed to comply with the recommendations in the guidelines in 80.75% of the sample. After multivariate analysis it was observed that patients with a higher level of education (OR = 0.24; CI95% 0.07-0.78), and those with a previous episode of AMI (OR = 0.18; CI95% 0.04-0.95) had a higher probability of using a therapy complying with the guidelines. These findings emphasized the importance of a Heart Team to develop a patient-directed educational tool to improve adherence to the treatment of coronary artery disease to patients.

In medical science, it is important to keep questioning established dogmas. For decades, the use of beta-blockers has been considered a cornerstone of medical therapy after ACS, having a class I or a class IIa indication for patients after STEMI and non-STEMI, respectively.^19,20^ However, in the era of reperfusion therapy, several studies have questioned this indication, especially in patients without left ventricular dysfunction.^21,22^ In the November issue of Rev Port Cardiol, Timoteo et al.^23^ published a new article about this topic which “adds more fuel to the fire” to the ongoing discussion. Using a single center registry, they have used propensity score analysis to evaluate the one-year outcome of patients treated with beta-blockers in a sample of 1520 post-ACS patients. They observed that beta-blocker use was an independent predictor of total mortality, including in patients with normal or mildly reduced ejection fraction. This analysis had some limitations. Although they have used propensity score matching, some caution is advised in the interpretation of these results because of residual confounding. Moreover, compliance with treatment and, more importantly, the reasons for not prescribing a beta-blocker could not be assessed in this study. Therefore, this study is important because it reinforces the urgent need to design a pragmatic clinical trial to reassess the effectiveness and safety of beta-blockers in the modern era of reperfusion therapy.

Also, in medical science, it is important to keep questioning the treatment effectiveness delivered to our patients. De Souza e Silva et al.^24^ studied the survival rate of ischemic heart disease adult patients treated with percutaneous coronary intervention (PCI), in the state of Rio de Janeiro (RJ), from 1999 to 2014, paid by the Brazilian public healthcare system (SUS). They showed data of 19,263 patients (61 ± 11 years old, 63.6% men), and survival rates of men vs. women in 30 days, one year and 15 years were: 97.3% (97.0-97.6%) vs. 97.1% (96.6-97.4%), 93.6% (93.2-94.1%) vs. 93.4% (92.8-94.0%), and 55.7% (54.0-57.4%) vs. 58.1% (55.8-60.3%), respectively. They also observed that the oldest age group was associated with lower survival rates in all periods; PCI with stent placement had higher survival rates than those without stent placement during a two-year follow-up, and women had a higher survival rate than men within 15 years after PCI. These findings performed in a real-world population may help physicians to make decisions regarding the indication of PCI, considering the benefits and risks observed with this procedure.

## Arrhythmias

Atrial fibrillation (AF) is the most common sustained arrhythmia, and a significant risk factor for stroke, heart failure and mortality.^25,26^ The SAFIRA study,^27^ recently published in the RPC journal, aimed to determine the prevalence and epidemiology of AF in a large sample of 7500 elderly Portuguese individuals. The study included a significant subset of 400 individuals that underwent 24-hour Holter monitoring and another subset of 200 individuals which had a 2-week event loop recorder to identify paroxysmal AF. Several interesting data came from this study. First, they observed a very high prevalence (9%) of AF in this elderly population, which was higher than previously reported.^28,29^ Second, more than one-third (35.9%) of AF patients were not aware of having the disease, and 18.6% had paroxysmal AF, which reinforces the need for more systematic AF screening.^30^ More importantly, in this “real-world” study the rates of anticoagulation were very disappointing. Although the mean CHADSVASC score was high (3.5 ± 1.2), most AF patients (56.3%) did not receive anticoagulation and only 25.8% were considered to be adequately anticoagulated. Therefore, this study highlights the enormous challenges in the diagnosis and management of AF in elderly patients and the urgent need to implement specific healthcare policies (involving patients, caregivers, doctors and health authorities) that can tackle these important problems.

As previously mentioned, the treatment of atrial fibrillation is a challenge in clinical practice especially with regard to the use of oral anticoagulants, which are fundamental for the prevention of stroke. Considering the challenges imposed by this sort of treatment, Stephan et al.^31^ hypothesized that mobile health support for shared decision-making may improve patients’ knowledge and optimize the decisional process. The authors developed an application (App aFib) to be used during the clinical visit, including a video about atrial fibrillation, risk calculators, explanatory graphics and information on the drugs available for treatment. In the pilot phase, 30 patients interacted with the application, which was evaluated qualitatively, and through a disease knowledge questionnaire and a decisional conflict scale. The number of correct answers in the questionnaire about the disease was significantly higher after the interaction with the application (from 4.7 ± 1.8 to 7.2 ± 1.0, p < 0.001), and the decisional conflict scale, administered after selecting the therapy with the app support, resulted in an average of 11 ± 16/100 points, indicating a low decisional conflict. Although these were initial findings, the App aFib improves patients` disease knowledge, and in the future newer studies may confirm if this finding could be translated into clinical benefit.

## Cardiovascular disease prevention and Epidemiology

The presence of cardiovascular risk factors in childhood creates a life-long burden which increases the risk of cardiovascular disease in adulthood.^32,33^ Therefore, several studies have showed the importance of evaluating risk factors and promoting healthy lifestyle across all lifespan, starting as early as in pre-school children.^34,35^ In an interesting study published in 2018, Melo Rodrigues et al.^36^ analyzed the prevalence and interrelation of cardiovascular risk factors in a sample of 1555 schoolchildren (6-9 years). First, they have found an enormous prevalence (29.1%) of overweight and obesity in this population, showing the magnitude of the childhood obesity epidemic.^37^ The prevalence of high-normal blood pressure (4.5%) and hypertension (3.7%) was also much higher than expected. There was a strong association between anthropometric body fat indicators and blood pressure, which reinforces the need for blood pressure measurement, in obese children. However, the most important take-home message from this study is to remember that our lifestyle behaviors as adults are linked to our previous exposures during childhood^31^ and, therefore, cardiovascular health promotion should involve all ages, starting from pre-school children, and the entire family.^38^

It is also known that lifestyle behaviors associated with an increased risk of cardiovascular disease are influenced by the individual’s health-related knowledge (health literacy) and by their perception of the risk of disease.^39^ Therefore, improving health literacy should be viewed as an essential tool to reduce the global burden of cardiovascular disease and improve risk factor control. In an innovative article published in 2018 in Rev Port Cardiol, Andrade et al.^40^ evaluated, in a large sample of 1624 portuguese individuals, the specific knowledge on cardiovascular disease, and its relationship with sociodemographic factors, health literacy and clinical history. It was striking to observe a major deficit in cardiovascular health-related knowledge. Only around one-third of the population was able to estimate their risk of myocardial infarction or stroke. Interestingly, participants identified non-smoking and a healthy diet as the main behaviors for cardiovascular disease prevention and attributed a lower importance to blood pressure control. It was also observed that only a very low percentage of individuals would call the national emergency number when faced with symptoms suggestive of a possible stroke or myocardial infarction, as also demonstrated in other studies.^41^ Therefore, this study clearly shows that there are important gaps in cardiovascular health-related knowledge in the general population. All of us, both as doctors, scientific community and society, need to create increasing awareness for the importance of improving health literacy in the community. This is a new and important strategy to help prevent cardiovascular disease.

It is fundamental to know about gaps in cardiovascular healthcare, and the knowledge about common problems and solutions shared by Portuguese-speaking countries (PSC) can provide us useful data regarding the similarities and differences between them, emphasizing well-succeeded actions for fighting CVD. Nascimento et al.^1^ described trends in cardiovascular disease morbidity and mortality in the PSC between 1990 and 2016, stratified by sex, and their association with the respective sociodemographic indexes (SDI) using the Global Burden of Disease (GBD) 2016 data and methodology. They observed large differences, mainly related to socioeconomic conditions, in the relative impact of CVD burden in PSC. Among CVD, ischemic heart disease was the leading cause of death in all PSC in 2016, except for Mozambique and Sao Tome and Principe, where cerebrovascular diseases have supplanted it. The most relevant attributable risk factors for CVD among all PSC are hypertension and dietary factors. Genetic factors, implicit in the cultural identity, factors inherent in the host, as well as the huge social inequality might have contributed to explain the mortality rates observed. Collaboration between the PSC might allow sharing successful experiences to confront CVD between those countries.

## Cardiomyopathies and Valvular heart disease

In the January 2018 issue of Rev Port Cardiol, Cardim et al.^42^ report the overall results of the Portuguese national Registry of Hypertrophic Cardiomyopathy (PRo-HCM), which included 1042 patients from 29 centers. This is one the largest and most significant worldwide registries of HCM, and provides a detailed contemporary assessment of the clinical profile, management strategies and outcomes of HCM in Portugal. The main conclusions were that HCM is characterized by relatively advanced age at diagnosis, with more than one fourth of patients diagnosed over the age of 65 years. There was a limited use of CMR for HCM assessment but, on the contrary, more than 50% performed genetic testing. The long-term mortality (0.65%/year) and the risk of sudden-cardiac death (0.22%/year) was low, but morbidity remained considerable. This registry shows that there are important differences in HCM management between guidelines and clinical practice, which was also demonstrated in other registries.^43,44^ This can be the result of different HCM clinical courses representing the heterogeneous spectrum of HCM. Finally, these data reinforce the importance of using clinical registries as an important source of information that should be used to inform practice but also to influence the writing of the guidelines.^45^

Advances in non-invasive cardiac imaging have provided important new insights in the pathophysiology of valvular heart disease and cardiomyopathies, and diagnosis of implanted device or bioprosthesis related complications.^46^ Gripp et al.^47^ used global longitudinal strain to assess the incidence of cardiotoxicity in 49 patients treated for breast cancer, and the independent factors associated with that event. Cardiotoxicity was identified in 5 (10%) on the third (n = 2) and sixth (n = 3) months of follow-up. Strain was independently associated with the event (p = 0.004; HR = 2.77; 95%CI: 1.39-5.54), with a cutoff point for absolute value of -16.6 (AUC = 0.95; 95%CI: 0.87-1.0) or a cutoff point for percentage reduction of 14% (AUC = 0.97; 95%CI: 0.9-1.0). They concluded that the 14% reduction in strain (absolute value of -16.6) allowed the early identification of patients who could develop anthracycline and/or trastuzumab-induced cardiotoxicity.

The role of incremental diagnostic and prognostic value of combination of imaging techniques or fusion imaging is growing exponentially.^48^ In the Valve Academic Research Consortium-2 (VARC-2) consensus document, quantitative and semi-quantitative hemodynamic assessments are recommended to assess aortic regurgitation (AR) severity by echocardiogram, and moderate-to-severe AR is defined as valve failure^48^ that is associated with poor outcome and mortality. Miyazaki et al.^49^ investigated a quantitative angiographic assessment of AR by videodensitometry before and after Balloon post-dilatation (BPD) since this technique provides an accurate assessment of the severity of paravalvular leak (PVL) and correlates with increased mortality and impaired reverse cardiac remodeling by echocardiography after transcatheter aortic valve implantation (TAVI). The authors showed that videodensitometry AR (VD-AR) decreased significantly from 24.0[18.0-30.5]% to 12.0[5.5-19.0]%, and the relative delta of VD-AR after BPD ranged from -100% (improvement) to +40% (deterioration). Significant AR (VD-AR > 17%) was observed in 47 patients (77%) before, and in 19 patients (31%) after BPD. They concluded that VD-AR after transcatheter heart valve implantation provides a quantitative assessment of post-TAVI regurgitation and can help in the decision-making process on performing BPD and in determining its efficacy.

The increasing number of children with evolving congenital heart diseases who had lower mortality, especially in recent years, demands greater preparation of professionals and institutions that handle them. Jatene surgery became the surgical procedure of choice to repair transposition of the great arteries (TGA) in neonates and infants, and nowadays the behavior of the neo-aortic valve is a cause of concern because of its potential for requiring late reoperation. Martins et al.^50^ assessed the prevalence and risk factors of neo-aortic valve regurgitation in 127 patients in the late postoperative period and observed 29% of mild and 18% of moderate neo-aortic valve regurgitation, in a long follow-up. Those patients had a higher aortic annulus Z-score, although reoperation rate due to neo-aortic regurgitation associated with aortic dilation was only 1.5%, all in patients with complex TGA group. So, this study shows that, despite the low incidence of reoperation after Jatene surgery, these patients require strict vigilance due to the time-dependent phenomenon, and one of the major risk factors for neo-aortic valve regurgitation was the preoperative pulmonary artery diameter.

## Cardiac function, exercise capacity and heart failure

Several studies have shown that left atrium (LA) size and function are important predictors of cardiovascular events in several clinical settings and can be involved in the progression to heart failure.^51-52^ In another interesting article published in Rev Port Cardiol, Fontes-Carvalho et al.^53^ evaluated in 94 patients after AMI the role of different indices of LA function (assessed by speckle tracking) as determinants of exercise capacity by cardiopulmonary exercise testing. They found a significant correlation between exercise capacity and LA conduit function, but not with contractile function. LA longitudinal strain was also associated with worse exercise capacity parameters, suggesting that this echocardiographic parameter can be used to predict reduced exercise capacity. Finally, it was shown that LA functional parameters were interdependent with LV diastolic function, highlighting the pathophysiologic importance of correct atrioventricular coupling. Therefore, this study highlights that although the LA was frequently viewed as a bystander in the regulation of cardiac function, the availability of new echocardiographic parameters for LA assessment (such as speckle tracking assessment) has shown its clinical utility as an important functional and prognostic marker in several clinical settings, especially in heart failure (HF).^54^

Heart failure patients have a significant risk of cardiovascular events. Therefore, several studies have tried to improve risk stratification tools to predict HF hospitalizations or the need for heart transplantation. The most commonly used score is the Seattle Heart Failure Model (SHFM), which is based on 24 clinical variables.^55^ Other scores are also available,^56^ but there is an ongoing need to improve risk stratification in HF. In the February issue of Rev Port Cardiol, Pereira-da-Silva demonstrate that VE/VCO2 slope, obtained from cardiopulmonary exercise testing (CPET), can be a good predictor of events in patients with HF with reduced EF (<40%). Although most previous studies have assessed the role of peak VO2 as a prognostic marker, it is known that the VE/VCO2 slope is a particularly interesting parameter, because it reflects ventilatory efficiency and is independent on the level of patient effort.^57^ The authors identified a threshold of VE/VCO2 slope > 39 as an excellent marker of worse outcome, with a c-statistic value of 0.79. Nevertheless, it is commonly said “in Medicine there are no magical numbers”. This is especially true in the selection of HF patients for heart transplantation, where individual clinical decision requires a team-based approach, with extensive clinical experience and a multiparametric approach. However, this interesting study highlights the importance of integrating the information provided by CPET, especially of VE/VCO2, as another important clinical parameter to better stratify these patients.

Exercise training induces cardiovascular adaptations secondary to changes in blood pressure, as well as other hemodynamic and metabolic changes in response to physical exertion that are most of the time desired by the cardiologist. Rodrigues et al.^58^ checked the effects of aerobic exercise training on contractility and intracellular calcium (Ca2+) transients of cardiomyocytes, and on the expression of microRNA 214 (miR-214) in the left ventricle of spontaneously hypertensive rats (SHR). They demonstrated that exercise training reduced systolic arterial pressure in hypertensive rats and increased the availability of intracellular Ca2+ by accelerating the sequestration of these ions in left ventricular myocytes of hypertensive rats, despite increased expression of miR-214 and maintenance of cell contractility. This study confirmed the anti-hypertensive effects of aerobic exercise, as already reported previously.

But will any level of exercise be beneficial to all? Silva et al.^59^ hypothesized that athletes engaging in high-intensity strength training for long periods of time show changes in cardiac structure associated with reduced cardiac function when compared to long-distance runners, and long-time exposure to high-intensity strength training could lead to a reduction of endothelial function caused by pressure overload. They evaluated 40 high-performance athletes (powerlifters [PG], n = 16; runners [RG], n = 24) and assessed heart structure and function performing echocardiography and checking systolic and diastolic blood pressure (SBP/DBP), flow-mediated dilation (FMD), peripheral vascular resistance (PVR), maximum force (squat, bench press, and deadlift), and maximal oxygen uptake (spirometry). The authors concluded that cardiovascular adaptations are dependent on training modality, and the borderline structural cardiac changes are not accompanied by impaired function in powerlifters. However, a mild increase in blood pressure seems to be related to PVR rather than endothelial function.

## Conclusions

We hope this review of the best in Cardiology and Cardiovascular Science published in the Portuguese language by 2 major journals can help our readers to update their knowledge in an easy and pleasant format and yet, get excited and interested in going deeper on the articles published last year on their field of expertise. The specific areas covered by this review included coronary artery disease, arrhythmias, cardiovascular disease prevention and epidemiology, cardiomyopathy and valvular heart disease, and finally cardiac function, exercise and heart failure. Articles published in all these fields demonstrated important innovation, new and original information with direct effect on clinical routine patient management, and also new insights on better understanding of disease process and treatment. Population and epidemiological data of particular importance for Portuguese speaking countries were also presented.

## References

[1] Nascimento BR, Brant LCC, Oliveira GMM, Malachias MVB, Reis GMA, Teixeira RA (2018). Epidemiologia das doenças cardiovasculares em
paísesde Língua Portuguesa: dados do "Global Burden of
Disease", 1990 a 2016. Arq Bras Cardiol.

[2] Fontes-Carvalho R, Gonçalves L (2018). The Portuguese Journal of Cardiology. Eur Heart J.

[3] Rochitte CE (2018). The New Impact Factor of the Arquivos Brasileiros de Cardiologia
(ABC Cardiol), 1.318: An Achievement of the SBC for Our Scientific
Community. Arq Bras Cardiol.

[4] Timóteo AT, Mimoso J, Registro Nacional de Síndromes Coronárias
Agudas (2018). Portuguese Registry of Acute Coronary Syndromes (ProACS): 15
years of a continuous and prospective registry. Rev Port Cardiol.

[5] Lawesson SS, Alfredsson J, Fredrikson M, Swahn E (2012). Time trends in STEMI - improved treatment and outcome but still a
gender gap:a prospective observational cohort study from the SWEDEHEART
registry. BMJ Open.

[6] Herrett E, Smeeth L, Walker L, Weston C, MINAP Academic Group (2010). The Myocardial Ischaemia National Audit Project
(MINAP). Heart.

[7] Pereira H, Calé R, Pinto FJ, Pereira E, Caldeira D, Mello S, Centers participating in the Stent for Life Initiative
Portugal (2018). Factors influencing the patient delay to primary angioplasty in
myocardial infarction with ST-segment elevation (STEMI): The Stent for life
initiative in Portugal. Rev Port Cardiol.

[8] Nallamothu BK, Normand SL, Wang Y, Hofer TP, Brush JE Jr, Messenger JC (2015). Relation between door-to-balloon times and mortality after
primary percutaneous coronary intervention over time: a retrospective
study. Lancet.

[9] De Luca G, Suryapranata H, Ottervanger JP, Antman EM (2004). Time delay to treatment and mortality in primary angioplasty for
acute myocardial infarction: every minute of delay counts. Circulation.

[10] De Luca G, Suryapranata H, Zijlstra F, van 't Hof AW, Hoorntje JC, Gosselink AT (2003). Symptom-onset-toballoon time and mortality in patients with acute
myocardial infarction treated by primary angioplasty. J Am Coll Cardiol.

[11] Neumann FJ, Sousa-Uva M, Ahlsson A, Alfonso F, Banning AP, Benedetto U (2018). 2018 ESC/EACTS Guidelines on myocardial
revascularization. Eur Heart J.

[12] Davies JE, Sen S, Dehbi HM, Al-Lamee R, Petraco R, Nijjer SS (2017). Use of the instantaneous wave-free ratio or fractional flow
reserve in PCI. N Engl J Med.

[13] Gotberg M, Christiansen EH, Gudmundsdottir IJ, Sandhall L, Danielewicz M, Jakobsen L, iFRSWEDEHEART Investigators (2017). Instantaneous wave-free ratio versus fractional flow reserve to
guide PCI. N Engl J Med.

[14] Cook CM, Jeremias A, Petraco R, Sen S, Nijjer S, Shun-Shin MJ (2017). Fractional flow reserve/instantaneous wave-free ratio discordance
in angiographically intermediate coronary stenoses: an analysis using
Doppler-derived coronary flow measurements. JACC Cardiovasc Interv.

[15] Menezes MN, Francisco AR, Ferreira PC, Jorge C, Torres D, Cardoso P (2018). Comparative analysis of fractional flow reserve and instantaneous
wave-free ratio: Results of a five-year registry. Rev Port Cardiol.

[16] Lee JM, Shin ES, Nam CW, Doh JH, Hwang D, Park J (2017). Clinical outcomes according to fractional flow reserve or
instantaneous wave-free ratio in deferred lesions. JACC Cardiovasc Interv.

[17] Kern MJ, Seto AH (2017). Is instantaneous wave-free ratio a new standard of care for
physiologic assessment of coronary lesions?More questions than
answers. Circulation.

[18] Bravo Baptista S, Raposo L (2018). Coronary pressure (sometimes) lies. Rev Port Cardiol.

[19] Farsky PS, Hirata MH, Arnoni RT, Almeida AFS, Issa M, Lima PH (2018). Persistent inflammatory activity in blood cells and artery tissue
from patients with previous bare metal stent. Arq Bras Cardiol.

[20] Borges JM, Almeida PA, Nascimento MM, Barreto Filho JA, Rosa MB, Sousa AC (2018). Factors associated with inadequate management of antiplatelet
agents in perioperative period of non- cardiac surgeries. Arq Bras Cardiol.

[21] Ibanez B, James S, Agewall S, Antunes MJ, Bucciarelli-Ducci C, Bueno H (2018). 2017 ESC guidelines for the management of acute myocardial
infarction in patients presenting with ST-segment elevation: the Task Force
for the management of acute myocardial infarction in patients presenting
with ST-segment elevation of the European Society of Cardiology
(ESC). Eur Heart J.

[22] Roffi M, Patrono C, Collet JP, Mueller C, Valgimigli M, Andreotti F (2016). 2015 ESC guidelines for the management of acute coronary
syndromes in patients pre-senting without persistent ST-segment
elevation. Eur Heart J.

[23] Dondo TB, Hall M, West RM, Jernberg T, Lindahl B, Bueno H (2017). β-Blockers and mortality after acute myocardial infarction
in patients without heart failure or ventricular dysfunction. J Am Coll Cardiol.

[24] Bangalore S, Steg G, Deedwania P, Crowley K, Eagle KA, Goto S (2012). β-Blocker use and clinical outcomes in stable outpatients
with and without coronary artery disease. JAMA.

[25] Timóteo AT, Rosa SA, Cruz M, Moreira RI, Carvalho R, Ferreira ML (2018). What is the role of beta-blockers in a contemporary treatment
cohort of patients with acute coronary syndromes? A propensity-score
matching analysis. Rev Port Cardiol.

[26] Silva CG, Klein CH, Godoy PH, Salis LH, Silva NA (2018). Up to 15-year survival of men and women after percutaneous
coronary intervention paid by the brazilian public healthcare system in the
state of Rio de Janeiro, 1999-2010. Arq Bras Cardiol.

[27] Kirchhof P, Benussi S, Kotecha D, Ahlsson A, Atar D, Casadei B (2016). 2016 ESC Guidelines for the management of atrial fibrillation
developed in collaboration with EACTS. Eur Heart J.

[28] Andersson T, Magnuson A, Bryngelsson IL, Frobert O, Henriksson KM, Edvardsson N (2013). All-cause mortality in 272,186 patients hospitalized with
incident atrial fibrillation 1995-2008: a Swedish nationwide long-term case
control study. Eur Heart J.

[29] Monteiro P (2018). The SAFIRA study: A reflection on the prevalence and treatment
patterns of atrial fibrillation and cardiovascular risk factors in 7500
elderly subjects. Rev Port Cardiol.

[30] Bonhorst D, Mendes M, Adragão P, De Sousa J, Primo J, Leiria E (2010). Prevalence of atrial fibrillation in the Portuguese population
aged 40 and over: the FAMA study. Rev Port Cardiol.

[31] Primo J, Gonçalves H, Macedo A, Russo P, Monteiro T, Guimarães J (2017). Prevalence of paroxysmal atrial fibrillation in a population
assessed by continuous 24-hour monitoring. Rev Port Cardiol.

[32] Freedman B, Camm J, Calkins H, AF-Screen Collaborators (2017). Screening for atrial fibrillation: A report of the AF-SCREEN
international collaboration. Circulation.

[33] Stephan LS, Almeida ED, Guimarães RB, Ley AG, Mathias RG, Assis MV (2018). Oral anticoagulation in atrial fibrillation: development and
evaluation of a mobile health application to support shared
decision-making. Arq Bras Cardiol.

[34] Biro FM, Wien M (2010). Childhood obesity and adult morbidities. Am J Clin Nutr.

[35] Williams CL, Hayman LL, Daniels SR, Robinson TN, Steinberger J, Paridon S (2002). Cardiovascular health in childhood: a statement for health
professionals from the committee on atherosclerosis, Hypertension, and
Obesity in the Young (AHOY) of the council on cardiovascular disease in the
young, American Heart Association. Circulation.

[36] Peñalvo JL, Santos-Beneit G, Sotos-Prieto M, Bodega P, Oliva B, Orrit X (2015). The SI! Program for cardiovascular health promotion in early
childhood: a cluster-randomized trial. J Am Coll Cardiol.

[37] Vedanthan R, Bansilal S, Soto AV, Kovacic JC, Latina J, Jaslow R (2016). Family-based approaches to cardiovascular health
promotion. J Am Coll Cardiol.

[38] Rodrigues PR, Pereira RA, Gama A, Carvalhal IM, Nogueira H, Rosado-Marques V (2018). Body adiposity is associated with risk of high blood pressure in
Portuguese schoolchildren. Rev Port Cardiol.

[39] Boelsen-Robinson T, Gearon E, Peeters A (2014). Incidence of childhood obesity in the United
States. N Engl J Med.

[40] Fuster V (2015). Stratified approach to health: integration of science and
education at the right time for each individual. J Am Coll Cardiol.

[41] Safeer RS, Cooke CE, Keenan J (2006). The impact of health literacy on cardiovascular
disease. Vasc Health Risk Manag.

[42] Andrade N, Alves E, Costa AR, Moura-Ferreira P, Azevedo A, Lunet N (2018). Knowledge about cardiovascular disease in
Portugal. Rev Port Cardiol.

[43] Pereira H, Pinto FJ, Calé R, Pereira E, Marques J, Almeida M (2014). Stent for life in Portugal: this initiative is here to
stay. Rev Port Cardiol.

[44] Cardim N, Brito D, Rocha LL, Freitas A, Araújo C, Belo A (2018). The portuguese registry of hypertrophic cardiomyopathy: Overall
results. Rev Port Cardiol.

[45] Cecchi F, Olivotto I, Betocchi S, Rapezzi C, Conte MR, Sinagra G (2005). The Italian registry for hypertrophic cardiomyopathy: a
nationwide survey. Am Heart J.

[46] Lipshultz SE, Orav EJ, Wilkinson JD, Towbin JA, Messere JE, Lowe AM, Pediatric Cardiomyopathy Registry Study Group (2013). Risk stratification at the time of diagnosis for children with
hypertrophic cardiomyopathy: a report from the Pediatric Cardiomyopathy
Registry Study Group. Lancet.

[47] Faxon DP, Burgess A (2016). cardiovascular registries: Too much of good
thing?. Circ Cardiovasc Interv.

[48] Delgado V, Knuuti J, Plein S, Achenbach S, Bax JJ (2018). The year in cardiology 2017: imaging. Eur Heart J.

[49] Gripp EA, Oliveira GE, Feijó LA, Garcia MI, Xavier SS, Sousa AS (2018). Global longitudinal strain accuracy for cardiotoxicity prediction
in a cohort of breast cancer patients during anthracycline and/or
trastuzumab treatment. Arq Bras Cardiol.

[50] Kappetein AP, Head SJ, Genereux P, Piazza N, van Mieghem NM, Blackstone EH (2013). Updated standardized endpoint definitions for transcatheter
aortic valve implantation: the valve academic research consortium-2
consensus document. J Thorac Cardiovasc Surg.

[51] Miyazaki Y, Modolo R, Abdelghani M, Tateishi H, Cavalcante R, Collet C (2018). Papel da avaliação aortográfica quantitativa
da regurgitação aórtica por videodensitometria na
orientação do implante da valva aórtica
transcateter. Arq Bras Cardiol.

[52] Martins CN, Gontijo Filho B, Lopes RM, Silva FD (2018). Mid- and longterm neo-aortic valve regurgitation after jatene
surgery: Prevalence and risk factors. Arq Bras Cardiol.

[53] Moller JE, Hillis GS, Oh JK, Seward JB, Reeder GS, Wright RS (2003). Left atrial volume: A powerful predictor of survival after acute
myocardial infarction. Circulation.

[54] Meris A, Amigoni M, Uno H, Thune JJ, Verma A, Køber L (2009). Left atrial remodelling in patients with myocardial infarction
complicated by heart failure, left ventricular dysfunction, or both: the
VALIANT echo study. Eur Heart J.

[55] Wong RC, Yeo TC (2010). Left atrial volume is an independent predictor of exercise
capacity in patients with isolated left ventricular diastolic
dysfunction. Int J Cardiol.

[56] Fontes-Carvalho R, Sampaio F, Teixeira M, Ruivo C, Ribeiro J, Azevedo A (2018). Left atrial deformation analysis by speckle tracking
echocardiography to predict exercise capacity after myocardial
infarction. Rev Port Cardiol.

[57] Blume GG, Mcleod CJ, Barnes ME, Seward JB, Pellikka PA, Bastiansen PM (2011). Left atrial function: physiology, assessment, and clinical
implications. Eur J Echocardiogr.

[58] Levy WC, Mozaffarian D, Linker DT, Sutradhar SC, Anker SD, Cropp AB (2006). The seattle heart failure model: prediction of survival in heart
failure. Circulation.

[59] O'Connor CM, Whellan DJ, Wojdyla D, Leifer E, Clare RM, Ellis SJ (2012). Factors related to morbidity and mortality in patients with
chronic heart failure with systolic dysfunction: the HF-ACTION predictive
risk score model. Circ Heart Fail.

[60] Guazzi M, Bandera F, Ozemek C, Systrom D, Arena R (2017). Cardiopulmonary exercise testing: what is its
value?. J Am Coll Cardiol.

[61] Rodrigues JA, Prímola-Gomes TN, Soares LP, Leal TF, Nóbrega C, Pedrosa DL (2018). Exercício físico e regulação de
cálcio intracelular em cardiomiócitos de ratos
hipertensos. Arq Bras Cardiol.

[62] Silva DV, Waclawovsky G, Kramer AB, Stein C, Eibel B, Grezzana GB (2018). Comparison of cardiac and vascular parameters in powerlifters and
long-distance runners: comparative cross-sectional study. Arq Bras Cardiol.

